# Bio-Environment-Induced Degradation and Failure of Internal Fixation Implants

**DOI:** 10.3390/jfb6041012

**Published:** 2015-10-15

**Authors:** Yan Zhou, Luke A. Perkins, Guodong Wang, Dongsheng Zhou, Hong Liang

**Affiliations:** 1 Materials Science and Engineering, Texas A&M University, College Station, TX 77843, USA; E-Mail: zhouyansdu@gmail.com; 2 Mechanical Engineering, Texas A&M University, College Station, TX 77843, USA; E-Mail: lukeallynperkins@gmail.com; 3 Department of Orthopedic Trauma, Shandong Provincial Hospital Affiliated to Shandong University, Jinan 250021, China; E-Mails: wanggd1986@163.com (G.W.); sdgkxh@aliyun.com (D.Z.)

**Keywords:** internal fixation, surface degradation, debris particles, titanium, stainless steel, surface roughness, microhardness, failure mechanisms, potentiodynamic polarization scans

## Abstract

Internal fixations provide fast healing but their failure remains problematic to patients. Here, we report an experimental study in failure of three typical cases of metals: a bent intramedullary stainless steel nail, a broken exterior pure Ti plate, and a broken intramedullary stainless steel nail. Characterization of the bent nail indicates that those metals are vulnerable to corrosion with the evidence of increased surface roughness and embrittlement. Depredated surface of the Ti plate resulted debris particles in the surrounding tissue of 15.2 ± 6.5 μm in size. Nanoparticles were observed in transmission electron microscope. The electron diffraction pattern of the debris indicates a combination of nanocrystalline and amorphous phases. The failure mode of the broken nail made of stainless steel was found to be fatigue initiated from the surface. This study clearly shows the biological-attack induced surface degradation resulting in debris and fatigue. Future design and selection of implant materials should consider such factors for improvement.

## 1. Introduction

Internal fixation is an innovation that is named by orthopedic surgeons as one of the most significant advances in fracture treatment. Those fixations shorten hospital stay, enable bones to heal fast, and reduce the number of improper healing or healing in improper position. Plates are used to hold pieces of fractured bones together with screws, and intramedullary nails are applied in fractures of long bones by fitting in medullary cavities. The repair of bone fractures follows three phases: inflammation phase, reparative phase (fibrocartilage callus formation and boney callus formation), and remodeling phase [1]. The dominant materials for internal fixation are mainly metals, include commercially pure Ti, Ti alloys, stainless steels, and Co–Cr alloys [2]. The interactions between a physiological environment and an internal fixation device limit the available materials. The device must be corrosion resistant in order to maintain its mechanical properties in the hostile and sensitive physiological environment, where blood and body fluid serve as excellent electrolytes [3]. The human body environment contains Cl^−^, bicarbonate, phosphate, K^+^, Na^+^, Mg^2+^, Ca^2+^, *etc.* [4] 316L stainless steel is one of the most commonly used steels for internal fixation devices that is an alloy of primarily Fe, Cr, and Ni [5]. The continuous oxide layer formed on the material surface protects the bulk material against corrosion. In the physiological environment, 316L is susceptible to oxide break-down and localized corrosion while Ti shows less corrosion [5]. The severe cases are caused by inflammation and even infection to the point that the devices must be removed. The tissue responds to implants and their debris by generating fibrosis layers. Minimum fibrosis formation is observed for Ti and thick fibrosis layers form around stainless steel, sometimes up to 2 mm [6]. The fibrosis tissues are detrimental to a rigid fixation between an implant and a bone. The debris also affects the bone resorption. By culturing human blood monocytes, Neale *et al.* reported that a decrease in bone resorption was caused by the metal particles, and Ti particles caused a less decrease than 316L [7].

Previous studies have mainly focused on the evaluation of wear particles of artificial hips and knee joints [8,9]. The internal fixation of bones is rarely brought into the scope of wear. Unlike the articular surfaces of implants that are optimized for tribological purposes, the fixation implants are designed for a long-time secure holding of fractured bones to facilitate healing. In artificial hips and knee joints, the wear debris particles from the rubbing surfaces trap in the joint capsule that fills with synovial fluids [10]. The wear debris from the fixation implants encounter muscle, bone surface, and bone marrow, where the internal environment is more complex and the potential detrimental effects on the body could be more severe. The size of wear debris generated from hip replacement was found to be 99 nm for Ti and 192 nm for Ti6Al4V [11].

Here, we report three cases of failed femoral fixation: a broken exterior plate of Ti TA2, a bent long intramedullary nail of stainless steel 316L, and a broken short intramedullary nail of stainless steel 316L. For the Ti plate, the wear debris embedded in tissues were focused and the tissue reaction was evaluated. To the best of our knowledge, no studies have been reported on the wear debris from an exterior plate for internal fixation. For the bent steel nail, the corrosive properties, surface roughness, and microhardness were examined. For the broken steel nail, the fractography and the microhardness were determined. The compressive analysis of a wide range of metallic failed implants sheds light on the field of orthopedic biomaterials.

## 2. Case Study and Discussion

### 2.1. Case I, Bio-environmental Attack

**Figure 1:**
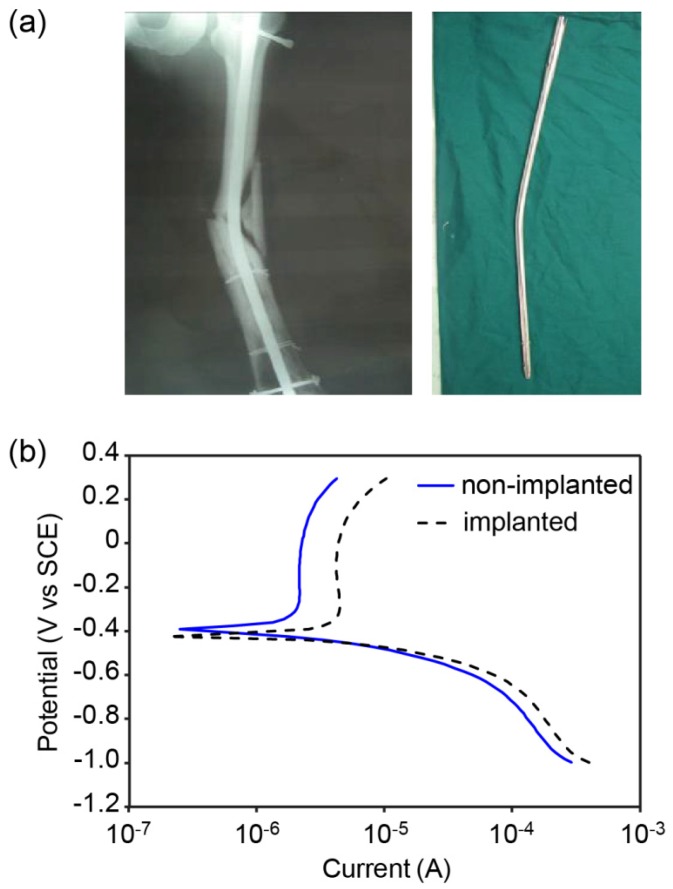
(**a**) The X-ray film and the extracted bent long intramedullary nail; (**b**) the potentiodynamic polarization scans of a non-implanted nail and the implanted nail.

Implants are sometimes damaged due to further accident. A bent long intramedullary nail of stainless steel 316L was collected during the revision surgery, which was performed four months after the initial surgery. The X-ray film shows a complicated fracture where the bone appears to be crushed into the intramedullary nail (Figure 1a). The potentiodynamic study reveals the corrosive behavior of the specimen. In Figure 1b, the passive region for the implanted material occurs at a much higher current than that for the non-implanted material. This implies an increased chance for corrosion on the implanted material if the two materials were placed in the same environment. The trend of the curve is typical for stainless steels.

**Figure 2:**
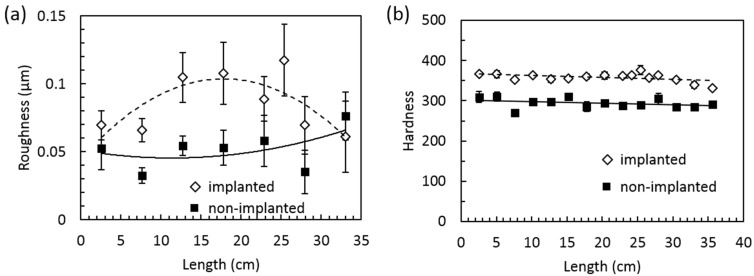
(**a**) The surface roughness of a non-implanted nail and the long implanted nail; (**b**) the HV microhardness.

There are several possible reasons. First, the bio-environment corrodes the metal surface. Second, the surface becomes rougher due to degradation. To verify this, we conducted surface roughness and hardness measurements to see if any degradation or hardening of the surface due to implanting. Figure 2 shows the results. Figure 2a is the roughness data comparing the implanted and otherwise. It is clear that the implantation makes the surface significantly rougher, meaning the detachment of the surface material. Correspondingly, the surface is hardened as well. This is the evidence of chemical reactions between the implant and physiological environment resulting in corrosion that leads to the increase of the surface roughness. The fact that the increased hardness might be due to the formation of oxide that is usually harder than metal itself, this is in line with the roughened surface. The increased surface roughness is a collective result of corrosion *in vivo*, the movement between the inner surface of the bone and the implant, and the mechanical collision during its surgical extraction.

### 2.2. Case II, Surface Degradation

**Figure 3:**
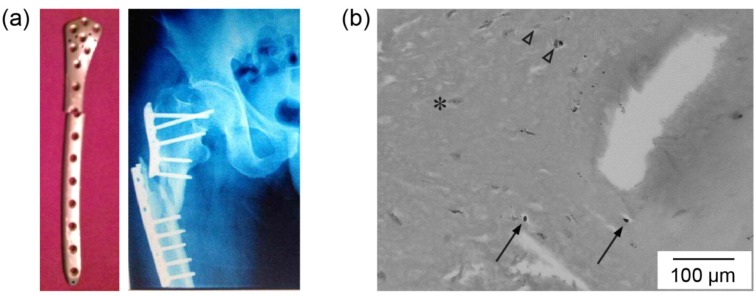
(**a**) The Ti exterior plate and the X-ray film of the patient; (**b**) the cross-section of the tissue collected from a hole of a locking screw (not on the broken site). Arrows point at the debris particles after encapsulation. Triangles point at the particles with surrounding inflammatory cells. Fibroblast is marked by an asterisk.

The biological environment-induced surface roughening is expected to lead to further degradation. Here is a case of such a scenario. Locking and unlocking screws are commonly used on an exterior plate. The Ti femoral plate broke on the position where a screw hole was (Figure 3a). The X-ray film of the patient shows a locking screw was originally installed. The residual stress from the screw increased the chance of failing of the plate. The tissue from another screw hole with a locking screw was obtained during the revision surgery. Debris of irregular shapes and varied sizes were observed in the tissue (Figure 3b). Some particles were only encapsulated by the surrounding tissue (marked by arrows) and others caused the presence of the inflammatory cells around them (marked by triangles), indicating the different fate of particles that might also be related to their encapsulation time. Fibroblasts play an important role in bone healing that were also seen (pointed by an asterisk). The images clearly show that debris particles generated from the minimal motion between the locking screw and the screw hole were later encapsulated by the tissue.

**Figure 4:**
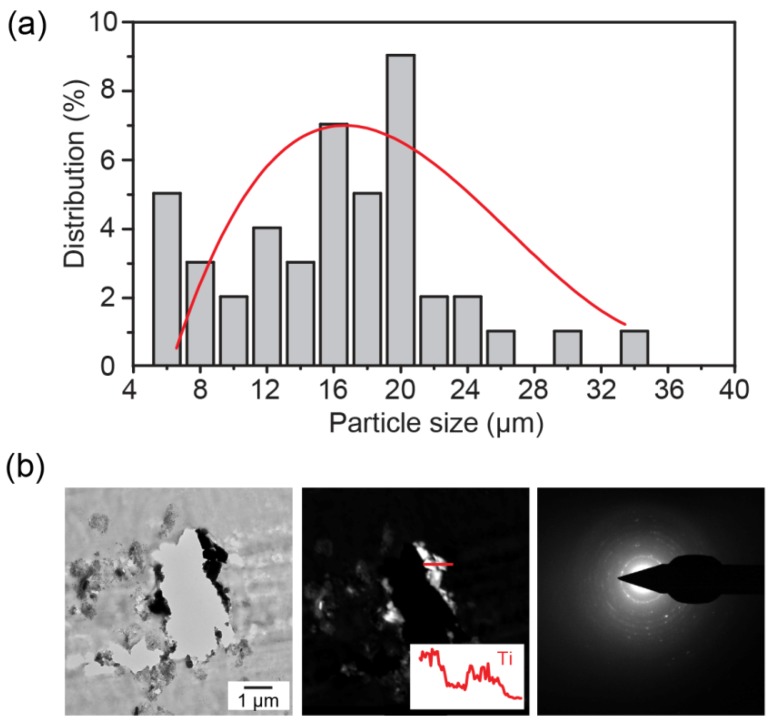
(**a**) Size distribution histogram of Ti debris particles with a polynomial fit; (**b**) the elemental confirmation of Ti particles embedded in tissue using TEM.

The size distribution and elemental information of the debris are shown in Figure 4. Micro-sized particles were counted based on the optical images. The Ti particle size is 15.2 ± 6.5 μm and the mode value is 20 μm, as shown in Figure 4a. The TEM image in Figure 4b confirms the existence of nano-sized debris particles that were undetectable by an optical microscope. The microtome cutting left a non-intact cross-section due to the fact that the razor was unable to cut through the metallic wear debris. The bright field and dark field TEM images are shown. The inset EDX pattern of Ti corresponds to the indicated line in the dark field image that confirms the particles were composed of Ti. The electron diffraction pattern on the right indicates a combination of nanocrystalline and amorphous phases in the debris.

### 2.3. Case III, Fatigue

**Figure 5:**
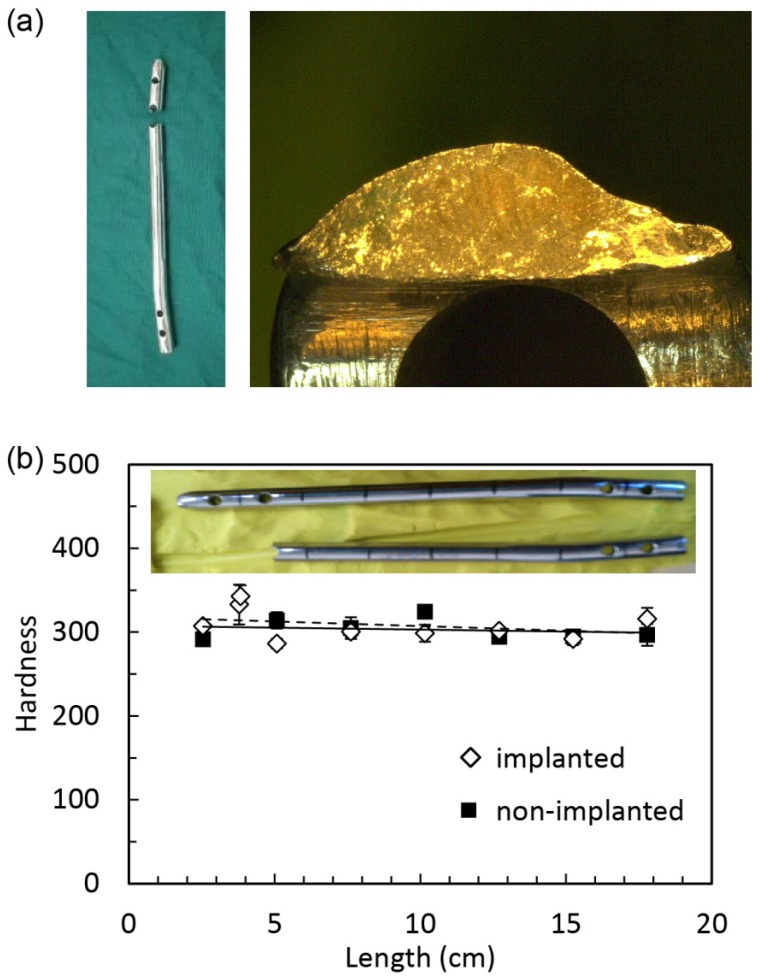
(**a**) The fractography of the broken short intramedullary nail of stainless steel; (**b**) the HV hardness shows that the surface of the implanted sample was harder around the fractured point.

Surface degradation provides enormous opportunity of origin for fatigue failure. An example was found in a short femoral intramedullary nail that displays brittle fracture due to fatigue, as seen in Figure 5a. The well-developed cracks were initiated at the surface. The fatigue occurred over a large number of cycles with stress at the fracture site well over the fatigue limit. Furthermore, the embrittlement was found on the surface close to the breaking point. As seen in Figure 5b, the hardness change was evidenced by the mechanical hardness tests. The implanted material was shown to be more brittle, which creates a condition for crack propagation rather than the stress distributing through ductile deformation. The hardness data show that the surface of the implanted sample was harder around the fracture point.

Due to the limited availability of implanted materials, in this research, we focus on three case studies that revealed information of critical failure modes in internal fixation implants that have not been reported. Although there is a wide variety of materials available for artificial joint implants [12,13,14], the internal fixations are dominantly metals. The environmental attacks from body fluids and other biological factors are critical factors for such materials. This study serves the purpose of identifying and understanding the failure mechanisms of those metals. Effort in collecting and understanding more and other types of materials will be made in future.

## 3. Experimental Procedure

### 3.1. Materials

The sample materials were provided by co-authors at the Shandong Provincial Hospital affiliated to Shandong University. Those implants failed inside the human body and were then extracted during the revision surgeries. The retrieved implants were sterilized in a high pressure steam sterilizer at 121 °C for 30 min. Identical non-implanted materials are used as control samples. The samples for exterior plates and nails are Ti TA2 and stainless steel 316L, respectively.

### 3.2. Gamry Reference 600 Potentiostat Machine and Equipment

Potentiodynamic polarization testing was performed using a Gamry Reference 600 potentiostat machine (Gamry Instruments, Warminster, PA, USA). A saturated calomel electrode (SCE) probe was used for a reference point. The approximate range of potential for stainless steel was determined from literature to be –1.2 V to 0.4 V. A solution of 2% NaCl in de-ionized water was used as the electrolyte. The concentration was chosen based on its corrosivity to most metals. For the samples, a one-inch portion was sawed off of the intramedullary nail. Tape was used to section off a specific area for testing. The exposed area was then measured to be 3.14 cm^2^. The test ran for approximately three minutes to span across the voltage range. The scan rate was 16 mV/s.

### 3.3. Particle Analysis

During the revision surgery, the tissue in a screw hole with a locking screw was collected and stored in 4% formaldehyde solution and later stained by hematoxylin and eosin (H&E stain), which was imaged using an optical microscope (VHX-600, Keyence Inc., Osaka, Japan). Software ImageJ (NIH, Bethesda, MD, USA) was applied for quantifying the size distribution of the wear particles that 50 of them were analyzed. Before H&E staining, a portion of the tissue was fixed and dehydrated for imaging by a transmission electron microscope (TEM, FEI Tecnai F20, Hillsboro, OR, USA). The TEM was operated at 200 kV and equipped with dark-field detector and an energy-dispersive X-ray spectroscopy (EDX) detector.

### 3.4. Surface Roughness and Micro-Hardness Analysis

The surface was analyzed using a 3D optical surface profiler (Zygo, Middlefield, CT, USA). A 10× objective was used for viewing the surface, and a 15 s scan elapsed during the interferometry process. The machine used in the micro-hardness measurements was a Vicker’s microhardness indenter (LM 300, LECO Corp., St. Joseph, MI, USA). The indenter was set to apply 0.3 kg.

## 4. Remarks

In summary, failure mechanics of metallic internal fixation devices have been explored through three case studies. The characterization on a bent intramedullary nail of stainless steel 316L shows that it has an increased chance for corrosion and an increased surface roughness and hardness, compared with a non-implanted device. The analysis of debris particles from a failed Ti exterior plate shows the size of debris particles is 15.2 ± 6.5 μm based on the optical imaging. Nanoparticles were also observed through TEM imaging. The electron diffraction pattern of the debris indicates a combination of nanocrystalline and amorphous phases. The failure mechanics of a broken intramedullary nail of stainless steel 316L is proposed to be brittle fracture due to fatigue. Through these cases it was found that the body’s environment poses bio-attack on implant metallic materials. The first indication is corrosion and surface roughening. Subsequently, the surface degrades with nano- and micro-particles. Under a cyclic stress due to daily activities, fatigue is generated eventually resulting in failure. As noted that all these can happen fairly quickly, within days and months. Detailed and more compressive analysis of a wide range of metallic failed implants are recommended that would shed light on the field of orthopedic biomaterials.
